# Longterm effects of palliative local treatment of incurable metastatic lesions in colorectal cancer patients

**DOI:** 10.18632/oncotarget.8090

**Published:** 2016-03-15

**Authors:** Qiong Yang, Fangxin Liao, Yuanyuan Huang, Chang Jiang, Shousheng Liu, Wenzhuo He, Pengfei Kong, Bei Zhang, Liangping Xia

**Affiliations:** ^1^ VIP Region, Sun Yat-sen University Cancer Center, Guangzhou, Guangdong, P.R. China; ^2^ Department of Oncology, Sun Yat-sen Memorial Hospital, Guangzhou, Guangdong, P.R. China; ^3^ State Key Laboratory of Oncology in South China, Sun Yat-sen University Cancer Center, Guangzhou, Guangdong, P.R. China; ^4^ Collaborative Innovation Center for Cancer Medicine, Guangzhou, Guangdong, P.R. China

**Keywords:** metastatic colorectal carcinoma, palliative local treatment, chemotherapy, propensity score matching, prognosis

## Abstract

We assessed the value of palliative local treatment of incurable metastatic lesions in colorectal cancer patients. Consecutive patients with metastatic colorectal cancer treated between 2003 and 2014 were retrospectively reviewed. Propensity score matching was used to create comparable palliative local treatment and chemotherapy alone groups (*n* = 272 in each group). The primary endpoint was overall survival, which was calculated using Kaplan-Meier survival analyses. Factors possibly influencing survival were evaluated by univariate and subsequently by multivariate analyses. Palliative local treatment prolonged survival as compared with chemotherapy alone (38.73 *vs*. 19.8 months, *p* < 0.01). Univariate and subsequent multivariate analyses showed that primary stage IV at initial diagnosis; high CA199 level and LDH at the time of diagnosis were independent factors for a poor prognosis. Palliative local treatment improved survival better than chemotherapy alone in patients with 0, 1, 2, or 3 of the prognostic factors (*p* < 0.01). Patients administered treatment for pulmonary metastases survived longer than those treated for metastases elsewhere (56.77 *vs*. 35.43 months, *p* = 0.01). Surgical treatment provided marginally longer survival than non-surgical treatment (44.87 *vs*. 35.43 months, *p* = 0.05). These findings suggest palliative local treatment has survival benefit for selected patients with incurable metastatic colorectal cancer.

## INTRODUCTION

Approximately 40%-50% of newly diagnosed colorectal cancer (CRC) patients have one or more metastatic lesions. Among those patients, only 33% have a chance for complete resection of the metastatic and primary lesions [1]. Complete resection, either initially or after chemotherapy, effectively improves overall survival (OS) [2-4]. By contrast, patients with incurable metastatic lesions have little chance of surviving over 5 years. For those patients, standard chemotherapy with monoclonal antibodies provides only 10.8 months of progression-free survival (PFS) and 29.9 months of OS [5, 6]. It is, therefore, necessary to explore new approaches to treat these patients.

The evolution of local treatment with curative intention reflects the fact that active surgery plays an important role in treatment of metastatic (m) CRC. For example, hepatic metastasectomy for mCRC was regarded as providing no chance for a cure two decades ago. Later, however, it was demonstrated that this procedure brings a survival benefit as long as patients the patient has fewer than four metastatic lesions with diameters less than 3 cm and with a 1cm clean surgical margin. And now, the requirements for this procedure have been simplified further to complete resection of hepatic lesions as long as adequate liver function is preserved after the operation [7-11].

Palliative local treatment as part of a treatment plan to eradicate some of the metastatic lesions has seldom explored in incurable mCRC except in response to an emergency, such as intestinal obstruction, bleeding or perforation. It was reported that palliative local treatment didn't improve long-term outcome for patients with widespread disease [12]. Nonetheless, recent studies suggest survival is improved in mCRC patients who undergo local treatment of metastatic lesions, as compared to historical controls administered chemotherapy alone [13-15]. But these studies have limitations, including a small number of patients and no control group or data from subgroup analysis. Consequently, the value of palliative local treatment for incurable mCRC remains unclear.

The aim of the present study, therefore, was to assess long-term survival in mCRC patients receiving palliative local treatment of incurable metastatic lesions and the factors affecting the outcome of this procedure.

## RESULTS

**Figure 1:**
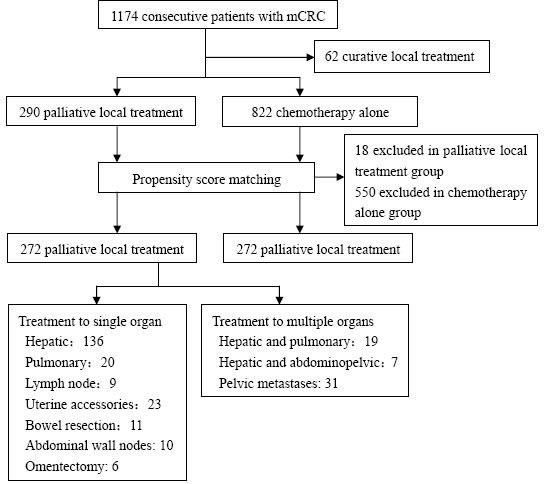
Flow chart of patient's inclusion and overview of palliative local treatment The variables used for matching included age, sex, primary location, stage at the first diagnosis, K-RAS status, and the number of metastatic lesions. After propensity score matching, the patients' baseline characteristics were balanced between palliative local treatment group and chemotherapy alone group.

As shown in Figure 1, 1174 consecutive mCRC patients were enrolled while 62 (5.3%) patients who received curative local treatment were excluded. Ultimately, 290 (24.7%) patients initially entered the palliative local treatment group, and 822 (70%) entered the chemotherapy alone group. Due to the imbalance in baseline data between the two groups, propensity score matching was used to minimize selection bias. The variables used for matching included age, sex, primary location, stage at first diagnosis, status of K-RAS, and the number of metastatic lesions. After propensity score matching, 544 patients were included, with 272 in each group. A median of 2 lines of chemotherapy were used in both the palliative local treatment (range 0-8) and chemotherapy alone (range 0-6) groups. Additional details of patients' characteristics are summarized in Table 1.

**Table 1:** Baseline demographics and clinical characteristicsof mCRCpatients before and after propensity score matching

Characteristics	Before matching (N)		*P*	After matching (N)		*P*
	Local treatment	Control		Local treatment	Control	
Total	290	822		272	272	
Sex			0.50			0.93
Men	182	534		173	174	
Women	108	288		99	98	
Age (years)			0.06			0.29
Median (range)	53 (13-80)	55(10-89)		53 (13-80)	53(10-80)	
<65	236	624		222	212	
≥65	54	198		50	60	
ECOG PS			<0.01			0.87
0	60	100		59	56	
1	220	678		206	205	
2	10	44		9	11	
Primary tumor site			<0.01			0.06
Colon	172	581		164	185	
Left-semicolon	108	320		104	119	
Right-semicolon	64	261		60	66	
Rectum	118	241		108	87	
Stage at initial diagnosis			<0.01			0.22
Metastatic disease	172	607		163	177	
Primary site resected	153	277		145	119	
Primary site unresected	19	230		18	58	
Non-metastatic disease	118	215	0.03	109	95	0.42
I	3	3		3	1	
II	44	56		41	30	
III	71	156		65	64	
Tumor differentiation (grade)			0.02			0.41
Well	15	33		14	13	
Moderate	175	412		175	183	
Poor	47	174		47	52	
Mucinous adenocarcinoma	36	127		36	24	
Unknown	17	76		0	0	
KRAS status			<0.01			0.18
Wild type	92	147		90	111	
Mutation type	25	60		24	21	
Unknown	173	615		158	140	
Number of metastatic lesions			<0.01			0.06
1	23	16		20	13	
2	20	10		15	10	
3	18	3		10	3	
4	8	3		7	3	
≥5	221	790		220	243	
Metastatic sites			0.01			0.32
Liver	76	254		72	85	
Lung	16	57		15	22	
Liver and lung	38	58		26	23	
Other	160	453		159	142	
Line of chemotherapy			<0.01			0.09
1	285	796		267	268	
2	206	425		201	168	
3	112	155		110	76	
≥4	48	61		46	31	
Systematic chemotherapy			0.11			1.00
Received	286	796		268	268	
Not received	4	26		4	4	
Target drugs			<0.01			0.42
Anti-VEGF / Anti-EGFR	145	277		140	133	
Neither	145	545		32	39	
Pre-treatment CEA (ng/ml)	350.35±90.1	451.65±69.24	0.48	359.91±94.1	427.13±115.06	0.66
Pre-treatment CA 199 (U/ml)	443.94±127.34	1361.75±205.52	<0.01	427.13±115.06	795.97±169.71	0.11
Pre-treatment LDH (U/L)	270.20±20.74	368.08±18.32	<0.01	270.38±21.49	332.09±24.14	0.06

Abbreviations: PS, performance status; VEGF,vascular endothelial growth factor; EGFR,epidermal growth factor receptor; CEA,carcinoembryonic antigen;CA199,carbohydrate antigen 199; LDH,lactate dehydrogenase.

### Survival benefit from palliative local treatment

**Figure 2:**
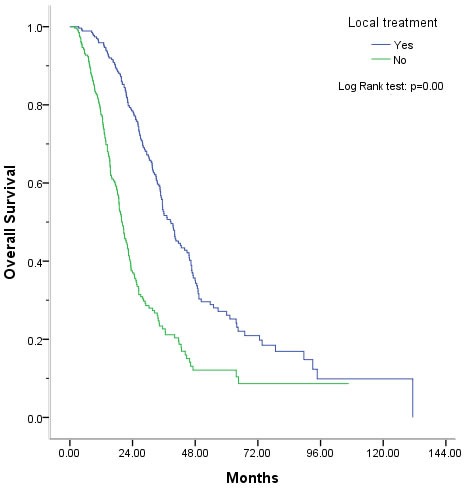
Overall survival benefit from adding palliative local treatment The median OS was 38.73 months in the palliative local treatment group *vs*. 19.8 months in chemotherapy group (*P* < 0.01). The corresponding 5-year survival expectancy was 26.2% and 11% in palliative local treatment group and chemotherapy alone group, respectively (*P* < 0.01).

By the last follow-up (median 53.3 months, range 1.5-142.0 months), tumors had recurred in 199 (73.2%) of the 272 locally treated patients. Among those, 61 (30.7%) were recurrences of previously treated lesions and 138 (69.3%) were new lesions, with or without recurrence of previously treated lesions. Eighty-six (43.2%) of the recurrences were treated with two or more repeats of palliative local treatment. Among the patients in the palliative local treatment group, 163 (59.9%) had died by the last follow-up and 109 (40.1%) were alive. In the chemotherapy alone group, 184 (67.6%) patients had died by the last follow-up and 88 (32.4%) were alive. The median OS in palliative local treatment and chemotherapy alone were 38.73 months (95%CI 34.93-42.54) and 19.8 months (95%CI 18.06-21.54), respectively (*P* < 0.01). The corresponding 2-, 3- and 5-year survival rates were 78%, 52.2% and 26.2%, respectively, in palliative local treatment group and 37%, 22% and 11%, respectively, in the chemotherapy alone group, (*P* < 0.01) (Figure 2).

### Who would be benefit from palliative local treatment?

There were 12 factors that could potentially influence OS, and 7 were significantly associated with outcome in a univariate analysis (Table 2). These included the primary tumor site (*P* = 0.02), stage at the first diagnosis (*P* < 0.01), tumor pathological grading (P = 0.004), lines of palliative chemotherapy (*P* < 0.01), metastatic sites (*P* < 0.01), level of CEA (*P* = 0.01), CA199 (*P* < 0.01), and LDH (*P* < 0.01) before all palliative treatment. Multivariate analysis (Table 3) showed that stage IV at the first diagnosis (*P* = 0.02), a pre-treatment CA199 level ≥35U/ml (*P* < 0.01) and a pre-treatment LDH level ≥ 245U/L (*P* = 0.003) were independent prognostic factors associated with poor OS. The 5-year survival rate in patients positive for 0, 1, 2, or 3 of those prognostic factors was 34.5%, 20.0%, 6.5%, and 0%, respectively (*P* < 0.01). Compared with chemotherapy alone, palliative local treatment improved survival whether the patient exhibited 0, 1, 2, or 3 of the prognostic factors (*P* < 0.01, details shown in Table 4).

**Table 2:** Predictors of overall survival based on univariate analysis

Factors	Number of patients	Median OS(95%CI) (months)	5-year SR (%)	*P*
Sex				0.48
Men	173	35.43 (30.83-40.04)	32.4%	
Women	99	39.70 (31.06-48.34)	17.7%	
Age (years)				0.75
<65	222	39.53 (33.41-45.66)	27.7%	
≥65	50	34.77 (28.04-41.49)	19.8%	
Primary tumor site				0.02
Colon	164	35.43 (31.38-39.49)	22.3%	
Rectum	108	42.67 (37.00-48.34)	31.7%	
Stage at initial diagnosis				<0.01
Metastatic disease	163	35.43 (31.25-39.62)	17.1%	
Non-metastatic disease	109	46.67 (38.03-55.31)	39.8%	
Tumor differentiation (grade)				0.004
Well	14	72.60 (55.87-89.33)	80.8%	
Moderate	175	39.50 (32.18-46.82)	26.1%	
Poor	47	33.40 (28.81-38.00)	12.5%	
Mucinous adenocarcinoma	36	36.03 (23.04-49.03)	20.7%	
KRAS status				0.37
Wild type	90	46.67 (39.72-53.62)	38.7%	
Mutation type	24	35.13 (29.74-40.53)		
Unknown	158	36.03 (31.42-40.64)	21.6%	
Number of metastases				0.15
1	20	40.60 (31.19-50.16)	35.2%	
2	15	34.77 (25.86-43.66)	15.4%	
3	10	64.43 (27.43-101.44)	53.3%	
4	7		77.1%	
≥5	220	36.00 (32.15-39.85)	22.4%	
Sites of local treatment				0.001
Liver	72	35.43 (31.05-39.82)	20.3%	
Lung	15	56.77 (32.62-80.92)	45.7%	
Liver and lung	26		65.6%	
Other	159	36.00 (30.38-41.62)	22.5%	
Line of chemotherapy				<0.01
1	267	37.23 (30.27-41.20)	35.1%	
2	201	31.47 (27.60-35.34)	16.3%	
3	110	46.37 (44.70-48.04)	21.2%	
≥4	46	46.67 (32.18-56.15)	38.3%	
Pre-treatment CEA				<0.01
Normal (<5ng/ml)	53	40.60 (33.36-47.83)	32.0%	
Abnormal (≥5ng/ml)	142	34.77 (29.95-39.59)	18.3%	
Not reported	77	42.67 (32.00-53.33)	35.1%	
Pre-treatment CA 199				<0.01
Normal (<35 U/ml)	92	43.93 (36.40-51.47)	28.3%	
Abnormal (≥35 U/ml)	89	31.60 (23.37-39.83)	9.6%	
Not reported	91	45.63 (34.78-56.49)	37.3%	
Pre-treatment LDH				<0.01
Normal (<245U/L)	133	39.53 (34.31-44.76)	25.8%	
Abnormal (≥245U/L)	45	31.80 (25.10-38.50)	8.0%	
Not reported	94	42.67 (32.09-53.24)	33.6%	

Abbreviations: OS, overall survival; SR, survival rate;CEA,carcinoembryonic antigen;CA199,carbohydrate antigen 199; LDH,lactate dehydrogenase.

**Table 3:** Predictors of survival based on multivariate analysis

Factors	Number of patients	HR (95%CI)	*P*
Stage at initial diagnosis	544	1.39 (1.05-1.85)	0.02
Pre-treatment CA199 ≥35U/ml	544	1.61 (1.24-2.09)	<0.01
Pre-treatment LDH ≥245U/L	544	1.50 (1.14-1.97)	0.003

Abbreviations: HR, hazard ratio; CA199, carbohydrate antigen 199; LDH, lactate dehydrogenase.

**Table 4:** Survival related to the number of prognostic factors in the local treatment and chemotherapy alone groups

Prognostic category	Groups	OS (95%CI) months	2-year SR	3-year SR	5-year SR	*P*
0	Local treatment	60.03 (41.52-78.55)	83.7	61.8	47.9	0.002
	Chemotherapy alone	29.07 (13.98-44.15)	51.2	29.8	14.9	
1	Local treatment	41.53 (37.38-45.69)	82.4	61.0	16.5	0.04
	Chemotherapy alone	25.73 (19.24-32.23)	50.4	36.7	26.7	
2	Local treatment	31.80 (24.69-38.91)	66.5	29.9	7.0	0.001
	Chemotherapy alone	19.47 (17.52-21.41)	27.2	18.7	6.1	
3	Local treatment	31.57 (17.93-45.20)	63.6	42.1	0	<0.01
	Chemotherapy alone	15.27 (13.93-16.61)	11.7	4.4	0	
Total	Local treatment	37.13 (33.27-41.00)	76.1	50.8	22.3	<0.01
	Chemotherapy alone	19.47 (17.85-21.09)	34.8	22.9	13.4	

Abbreviations; OS, overall survival; SR, survival rate.

### When is the best time to administer palliative local treatment?

**Figure 3:**
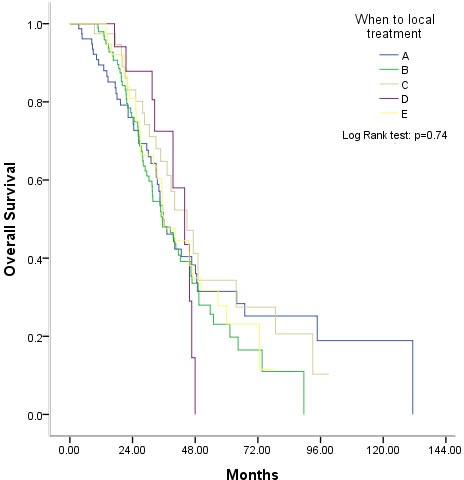
Effect of the timing of palliative local treatment on overall survival **A.** Before the first-line therapy, **B.** During the first-line therapy, **C.** After the first-line therapy and before the second-line therapy, **D.** During the second-line therapy, E, After the second-line therapy.

**Figure 4:**
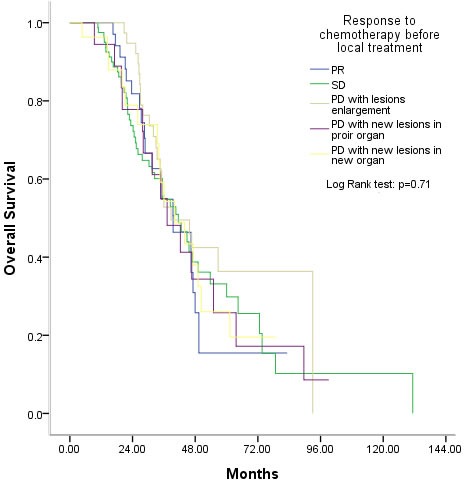
Overall survival after palliative local treatment was unrelated to the response to chemotherapy before palliative local treatment PR, partial response, SD, stable disease, PD, progressive disease.

Of the 272 patients administered palliative local treatment, 77 received it before palliative systemic chemotherapy, 99 received it during first-line chemotherapy, 40 after first-line chemotherapy but before the second-line chemotherapy, 17 during the second-line chemotherapy, and 39 after the second-line chemotherapy. The timing of the administration of palliative local treatment had no effect on survival benefit (*P* = 0.74, Figure 3). In addition, whether there was a response to chemotherapy before local treatment also had no effect on survival benefit (*P* = 0.71, Figure 4).

### What is the most effective type of palliative local treatment?

**Figure 5:**
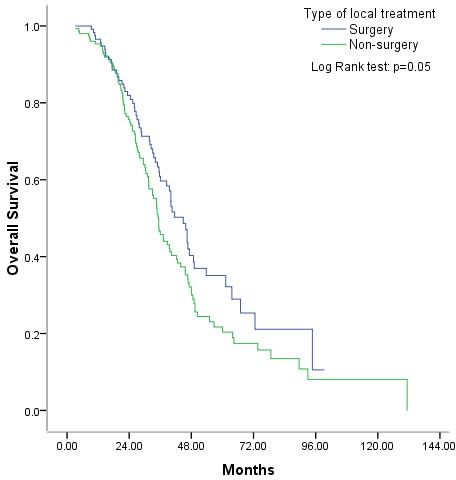
Overall survival was marginally better with surgical than non-surgical palliative local treatment

**Figure 6:**
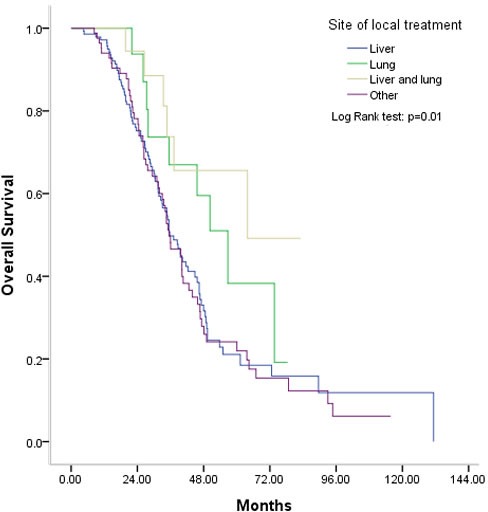
Overall survival was better after palliative local treatment of pulmonary metastases than other metastatic sites

Of those administered palliative local treatment, 120 were treated surgically to remove metastases (metastasectomy), while 152 were non-surgically. The nonsurgical treatments included RFA, PMCT, radioactive particle implantation, and radiation. The median OS was 44.87 months in the metastasectomy group and 35.43 months in non-surgery group (*P* = 0.05) (Figure 5). Patients who underwent palliative local treatment for pulmonary metastases had a median OS of 56.77 months, which was longer than those with hepatic or other metastases (median OS, 35.43 months, *P* = 0.01) (Figure 6).

## DISCUSSION

Palliative local treatment is increasingly being investigated for use with incurable metastatic lesions in mCRC because it may have survival benefit. For example, palliative thoracic radiotherapy is being used to treat advanced stage small cell lung cancer patients [16], and palliative local treatment is being administered to isolated progressing lesions in non-small cell lung cancer [17]. Using propensity score matching, we confirmed that adding palliative local treatment improves survival in patients with mCRC.

There are three important differences between the present study and earlier ones. First, 1112 consecutive eligible patients treated between 2003 and2014 were included. To the best of our knowledge, this was the largest study to systematically explore the value of palliative local treatment in patients with unresectable mCRC. Second, patients receiving local treatment may have an expectation of a longer life, and good general condition as well as a desire for active treatment. Therefore, to minimize selection bias, this study set chemotherapy alone as the control group and used propensity score matching to make the two groups comparable [18]. Third, patients included in the present study all had highly advanced mCRC. Over 80% had more than 5 metastatic lesions, and 66.5% had two more metastatic sites. By contrast, in Ferguson et al study and Hsu et al study, respectively, only 30% and 33.3% of patients had extrahepatic metastases at the time RFA was administered to the hepatic lesions [14, 15].

We also explored who would most benefit from this procedure? In general, patients in poor condition or with poorer prognosis do not benefit from palliative local treatment. Our univariate and multivariate analyses indicate that patients had poorer survival if they had one or more of these factors: primary stage IV at the first diagnosis, a pre-treatment CA199 level ≥ 35U/ml [19-21] and a pre-treatment LDH ≥ 245U/L [22-24]. When the patients were divided based on their exhibiting 0, 1, 2, or 3 of those factors, the corresponding 5-year OS rates were 34.5%, 20.0%, 6.5% and 0%, respectively. Further analysis was done to determine which patients would most benefit from palliative local treatment. Somewhat surprisingly, all four groups showed improved survival, though only patients with 0 or 1 prognostic factor had much longer survival (median OS > 40 months). In other words, it may be appropriate for palliative local treatment to be administrated to patients with a better prognosis.

When and how to perform the palliative local treatment was also investigated, but no correlation was found between survival benefit and the timing of local treatment or the response to chemotherapy. Surgical treatment produced marginally longer survival than non-surgical treatment. However, that result is contradicted in patients only treated with curative resection of liver metastasis after downstaging using neoadjuvant chemotherapy [1]. This reflects the nature of palliative local treatment, the aim of which is to control new lesions or reduce their potential impact on organ functions, not eradicate the tumor. On the other hand, it was not expected that palliative local treatment of pulmonary metastases would provide longer OS than treatment of other metastatic sites. Lung metastases are significantly less responsive to chemotherapy and correlate with poor survival in mCRC [25]. However, Kobayashi et al suggest that among patients with simultaneously detected metastases, metastasectomy appears to be beneficial only in patients with limited pulmonary disease [26]. In addition, Nagakura et al. reported that patients who undergo pulmonary resection alone survive longer than those who undergo hepatectomy alone, though the difference was not significant [12]. This suggests pulmonary metastases should be treated more actively in future.

There are inevitable limitations to the present study, particularly its retrospective nature, the long inclusion period, and missing data on tumor biomarkers and K-ras status. We tried our best to reduce bias by making the treatment decisions through a multidisciplinary team (MDT) model [27]. But further studies will be needed to address those issues.

In sum, the present study demonstrates that a survival benefit is obtained from palliative local treatment of incurable metastatic lesions in mCRC patients. Although the survival benefit did not correlate with prognosis, patients with a better prognosis obtained the greatest benefit. Well-designed prospective clinical trials will be needed to validate these results.

## MATERIALS AND METHODS

### Selection of the study population

Between January 1, 2003, and September 30, 2014, consecutive patients with histologically confirmed synchronous or metachronous mCRC treated at Sun Yat-sen University Cancer Center were retrospectively reviewed. The palliative local treatment included surgery, radiofrequency ablation (RFA), percutaneous microwave coagulation therapy (PMCT), radioactive particle implantation, and radiation. Prior to palliative local treatment, the patients were expected to have (1) ECOG performance scores of 0, 1, or 2; (2) adequate hepatic function [bilirubin < 2.0 mg/dl, transaminases levels < 3 times the normal upper limit (5 times for patients with liver metastasis)]; (3) adequate renal function (creatinine < 1.5 mg/dl); (4) adequate bone marrow function [absolute neutrophil count (ANC) >1,500/μl, hemoglobin >9.0 g/dl, and platelets >75,000/μl]; (5) a life expectancy of > 3 months. Exclusion criteria included curative local treatment, TACE, hepatic arterial infusion, non therapeutic exploratory laparotomy, emergency surgery for obstruction, hemorrhage and perforation, biliary drainage, and radiotherapy to treat bone metastasis for pain relief. The remaining patients were set as the chemotherapy alone group. The characteristics of patients in both groups were summarized in Table 1. The Institutional Review Board of the Sun Yat-sen Cancer Center approved this retrospective study.

### Systemic treatment

All patients received standard palliative chemotherapy unless the patient refused. The first- and second-line regimens included oxaliplatin-containing or irinotecan-containing chemotherapy with or without target drugs. The third- and later-line therapies had no mandatory requirement. The dosage, delivery, and schedule of main therapeutic regimens were based on the principles of the NCCN guidelines (version 3, 2015).

### Principles of palliative local treatment

At our center, whether palliative local treatment was administered, as well as related issues such as which lesions to treat, and the timing of intervention, was discussed by the MDT. Patients were considered suitable for palliative local treatment in the following settings: (1) liver or lung disease was predominantly limited, with or without minor additional disease; (2) most lesions were well controlled, with isolated new lesions or less than five enlarging lesions; or (3) metastatic lesions with a high risk of causing obstruction or oppression if they enlarge again, though patients had a partial disease response or stable disease during or after chemotherapy. Patients were allowed to receive one or more types or multiple administrations of palliative local treatments. Written informed consent was required before palliative local treatment.

### Statistical analysis

The primary endpoint was OS. All factors likely to correlate with prognosis were evaluated. To avoid selection bias between the two groups, propensity score matching was used to choose the cases for each group. Continuous variables are presented as the mean ± standard error, except for survival, which is presented as median (95 percent confidential interval, 95%CI). Independent-sample t tests were used for statistical analysis of continuous variables, and Fisher's exact test and χ2 analysis were used, as appropriate, for categorical data. All factors possibly influencing survival were evaluated using univariate and, subsequently, multivariate analyses. OS was calculated using the Kaplan-Meier method and data were compared using the log-rank test. Values of P less than 0.05 were considered statistically significant. Multivariate analysis using a Cox model was completed for all factors with a P value less than 0.05 in the univariate analysis. R version 2.8 was used for propensity score matching. SPSS version 18.0 was used for statistical analysis.
